# PTEN signaling is required for the maintenance of spermatogonial stem cells in mouse, by regulating the expressions of PLZF and UTF1

**DOI:** 10.1186/s13578-015-0034-x

**Published:** 2015-07-28

**Authors:** Wei Zhou, Hongfang Shao, Di Zhang, Jian Dong, Wei Cheng, Lu Wang, Yincheng Teng, Zhuo Yu

**Affiliations:** Hongqiao International Institute of Medicine, Shanghai Tongren Hospital / Faculty of Basic Medicine, Shanghai Jiao Tong University School of Medicine, Shanghai, 200025 China; Key Laboratory of Cell Differentiation and Apoptosis of Chinese Ministry of Education, Shanghai Jiao Tong University School of Medicine, Shanghai, 200025 China; Centre for Reproductive Medicine, Shanghai Jiao Tong University Affiliated Sixth People Hospital, Shanghai, 200233 China; Institute of Genetics and Developmental Biology, Chinese Academy of Sciences, Beijing, 100101 China; College of Public Health, Shanghai Jiao Tong University School of Medicine, Shanghai, 200025 China

**Keywords:** *Pten* knockout, PLZF, UTF1, Spermatogonial stem cells, PI3K-Akt signaling

## Abstract

**Background:**

*Pten* plays a crucial role in the stem cell maintenance in a few organs. *Pten* defect also causes the premature oocytes and ovary aging. We and other groups have found that the phosphatidylinositol-3-OH kinase (PI3K)-Akt signaling regulates the proliferation and differentiation of spermatogonial stem cells (SSCs). PTEN functions as a negative regulator of the PI3K pathway. Thus, we thought that the fate of SSCs might be controlled by *Pten*.

**Results:**

We report that promyelocytic leukaemia zinc finger (PLZF) and undifferentiated embryonic cell transcription factor 1 (UTF1), both of which are germ cell-specific transcriptional factors, are regulated by *Pten*. Conditional deletion of *Pten* leads to reduction in PLZF expression but induction of UTF1, which is associated with SSCs depletion and infertility in males with age.

**Conclusion:**

Our data demonstrate that *Pten* is required for the long-term maintenance of SSCs and precise regulation of spermatogenesis in mouse. The finding of a *Pten*-regulated GFRα1^+^/PLZF^−^/UTF1^+^ progenitor population provides a new insight into the precise mechanisms controlling SSC fate.

**Electronic supplementary material:**

The online version of this article (doi:10.1186/s13578-015-0034-x) contains supplementary material, which is available to authorized users.

## Background

Stem cells are capable of renewing themselves to maintain a stem cell pool as a preserved cell source for tissue homeostasis, while they can also differentiate into mature cells to carry out the function of a specific tissue. The precise balance of self-renewal and differentiation of stem cells is critical for the maintenance and function of a tissue or organ throughout life-time. Similar to other stem cells, spermatogonial stem cells (SSCs) renew themselves and meanwhile undergo a dramatic differentiation process-spermatogenesis to generate a large number of sperms consistently. Prior to spermatogenesis, multiple mitotic divisions of SSCs produce subpopulations of SSCs, and the balance of the SSC subpopulations is critical for long-term sperm production. Multiple proteins, such as promyelocytic leukaemia zinc finger (PLZF), GDNF family receptor alpha-1 (GFRα1) and undifferentiated embryonic cell transcription factor 1 (UTF1), are expressed in SSC subpopulations, which plays a crucial role in the maintenance of SSC pool. PLZF and GFRα1 are required in germ cells for stem cell self-renewal [1–3], whereas UTF1 is restricted to a small subset of spermatogonia that make the cells maintain the ability of differentiation [4, 5].

PTEN signaling is critical in governing the stem cell pool not only in the blood system and central neural system but also in reproductive system [6–8]. The loss of *Pten* in ovary via conditional knockout triggers premature of oocytes and ovary aging [8]. On the other hand, we and other groups have revealed that the phosphatidylinositol-3-OH kinase(PI3K)/Akt/S6 pathway is a critical signaling in controlling the proliferation and division of SSCs. Disruption of this signaling or *Akt* knockout leads to the loss of spermatogonial cells and infertility in males [9, 10]. PTEN is a major negative regulator of PI3K signaling [11, 12]. To understand the function of *Pten* in regulating SSC fate and fertility in male mouse, we generated conditional *Pten* knockout males using germ cell specific Cre strain, the *Stra8*-Cre mouse. It was turned out that the loss of *Pten* caused reduction of PLZF expression, but induction of UTF1. Thus, conditional *Pten* knockout leads to depletion of SSC pool and infertility with age.

## Results

### Conditional deletion of *Pten* in spermatogonial cells in mice

**Fig. 1:**
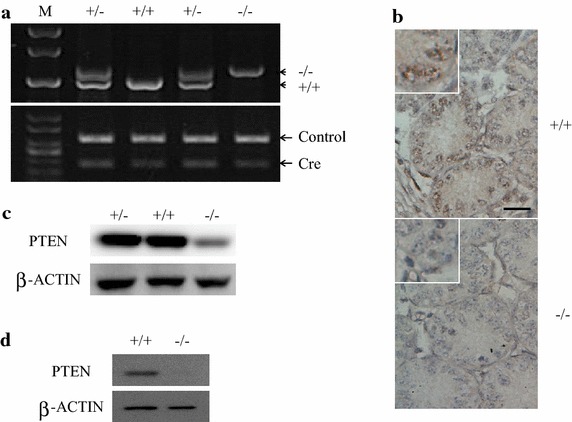
Cre-recombinase mediated deletion of *Pten* in SSCs. **a** PCR analysis of genotype of *Pten* knockout (KO) mice. *Short arrows* denote the predicted size of *Pten* KO and wild-type (WT) alleles, *long arrows* denote the predicted size of Cre and internal positive control (+/+, wild type; +/−, heterozygote; −/−,homozygote; M, Marker). **b** Immunostaining of PTEN in cross sections of 7 day-old testis. The cytoplasmic staining was observed in spermatogonial cells from *Pten* ^+/+^ section, whereas it was absent in the *Pten* ^−/−^ section (*scale bar* is 20 µm). **c** Western blot analysis of PTEN expression in whole testis of 32 day-old *Pten* ^+/+^, *Pten* ^+/−^, and *Pten* ^−/−^mice. PTEN is also expressed in nongerminal cells, so PTEN expression was detected in *Pten* ^−/−^ testes, but was significantly lower compared with *Pten* ^+/+^ and *Pten* ^+/−^ testes. **d** Absence of PTEN expression in haploid cells from *Pten* ^−/−^ testes. Haploid sperms were isolated from 79 day-old testes of *Pten* ^+/+^ and *Pten* ^−/−^ mice separately by sorting using flow cytometer, then were subjected to Western blotting analysis.

STRA8 is a germ-cell-specific protein and is expressed through neonatal spermatogonial cells to meiotic cells [13]. In the *Stra8*-EGFP transgenic mice, spermatogonial stem cells can be labeled by EGFP as characterized by transplantation assay [14]. Therefore, *Stra8*-Cre can be applied to generate SSC-specific gene knockout model. We created SSC-*Pten* null mice by crossing *Pten*^LoxP/LoxP^ mice with *Stra8*-Cre mice. The *Pten* knockout genotype was identified by examining the genomic allele of *Pten* (Fig. 1a), the *Pten* expression in testis sections of 7 day-old males (Fig. 1b) as well as in whole proteins from adult testes (Fig. 1c). Since *Pten* is also expressed in non-germ cells, we obtained purified haploid spermatids through cell sorting from adult mice to confirm the absence of *Pten* expression in germ cells from crossed mice (Fig. 1d).

### Conditional deletion of *Pten* caused overgrowth of testes followed by shrinking and sterility with age

**Fig. 2:**
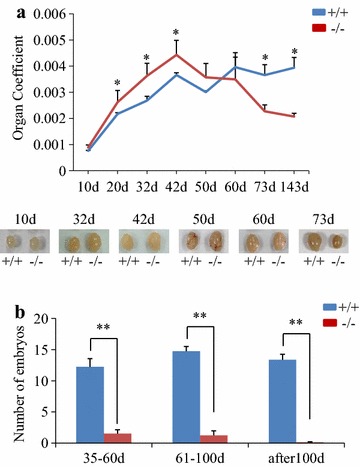
Overgrowth of testes and fertility loss in *Pten* ^−/−^ mice. **a** Curve of organ coefficient of *Pten* ^+/+^ and *Pten* ^−/−^ testes with age. *Horizontal bars* indicate mean values, n = 3, * *P* < 0.05. **b** In vivo fertility assay of *Pten* ^+/+^ and *Pten* ^−/−^ mice. The mating test was divided into three groups according to the male’s age: 35–60 days old, 61–100 days old, and over 100 days old. *Horizontal bars* indicate mean values, n = 22 ** *P* < 0.01.

**Fig. 3:**
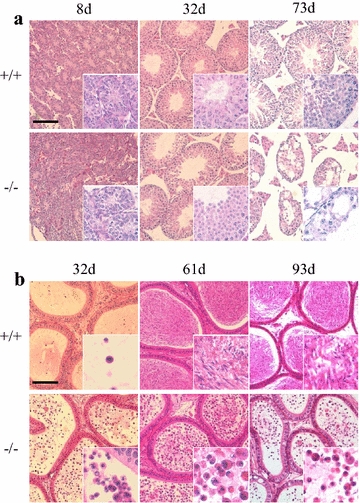
Abnormal spermatogenesis in *Pten* ^−/−^ mice. **a** Hematoxylin and eosin staining of sections from *Pten* ^+/+^ and *Pten* ^−/−^ testes at 7, 32 and 73 days old (*scale bar* is 100 µm). **b** Hematoxylin and eosin staining of epididymis sections from *Pten* ^+/+^ and *Pten* ^−/−^ mice at 32, 61, and 93 days old. Massive lack of normal sperm was observed in the epididymis of *Pten* ^−/−^ mice (*scale bar* is 100 µm).

The males with *Pten*-deleted in SSCs were grossly normal through all ages except that the size of testes was of overgrowth within the first 50 days after birth then shrunk afterwards (Fig. 2a). Fertility was found lower in the young *Pten*-deleted males and it was lost progressively after 60 days as measured by mating with wild type females (Fig. 2b). The morphology of testicular tubules cross-section was abnormal as showing larger lumen and failure of spermatogenesis with age (Fig. 3a). Furthermore, the rate of spermatogenesis was found very low in the *Pten*-deleted males during young ages, and severe loss of mature sperm production in the adult males epididymis after age of 60 days (Fig. 3b). This overall phenotype is very similar to that of *Pten* knockout in bone marrow stem cells and central neural stem cells as over-expansion of short-term hemeatopoietic stem cell pool and enlarged brain [6–8]. Possibly, the loss of *Pten* might disturb the balance of self-renewal and differentiation and promote excessive differentiation-associated proliferation of SSCs, thereby giving rise to enlarged testes in young mice but causing the depletion of stem cell source and infertility with age.

### Loss of *Pten* led to reduction of SSCs in neonatal males

**Fig. 4:**
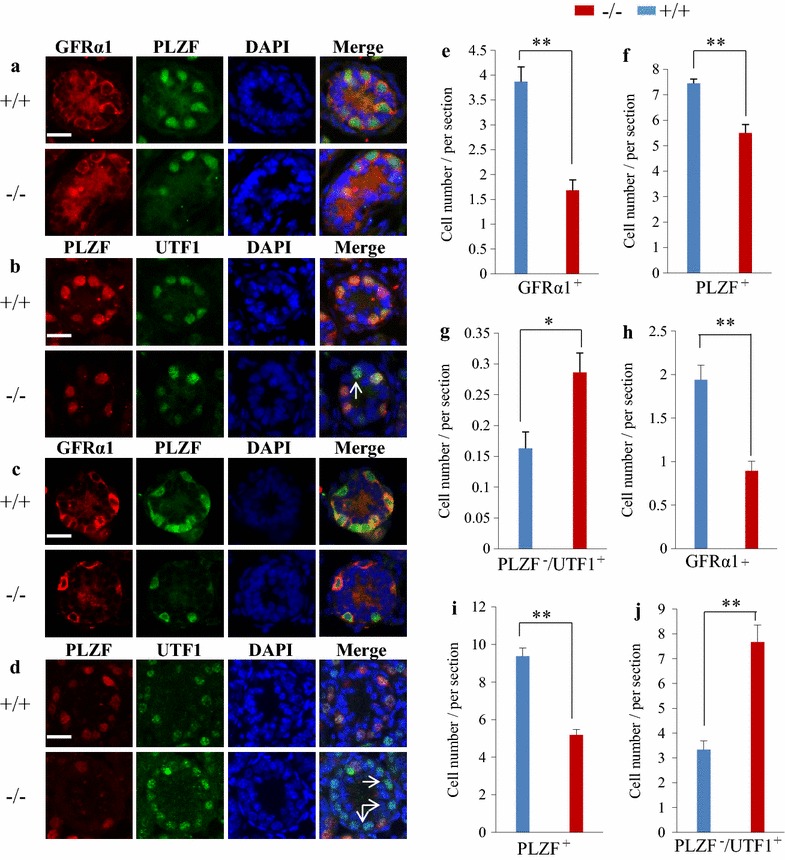
Reduction of GFRα1 and PLZF positive SSCs and increase of UTF1 positive cells in neonatal *Pten* ^−/−^ testes. Staining of 7 day-old testis sections showed the reduction of both GFRα1^+^ SSCs and PLZF^+^ SSCs **a** and the emergence of PLZF^−^/UTF1^+^ SSCs in the *Pten* ^−/−^ testis (**b**, *arrow*). Staining of 10 day-old testis sections showed a significant loss of both GFRα1^+^ SSCs and PLZF^+^ SSCs (**c**) and an increase in the number of PLZF^−^/UTF1^+^ cells in the *Pten* ^−/−^ testis (**d**, *arrows*). Nuclei were counterstained with DAPI (*scale bar* is 20 µm). Based on coimmunofluorescent staining of testicular tubule sections from both *Pten* ^+/+^ and *Pten* ^−/−^ mice at 7 days old (**e**–**g**) and at 10 day olds (**h**–**j**), the average number ± SEM of GFRα1^+^, PLZF^+^ and PLZF^−^/UTF1^+^ cells per tubule cross-section were calculated and presented as *bar graphs*. For the statistical analysis, each five inconsecutive testis sections were counted. *Horizontal bar* indicates mean value, n = 4, *P < 0.05, **P < 0.01.

To examine whether the self-renewal of SSCs was influenced by the absence of *Pten*, we used testis cross sections from 7 day-old pups and calculated the number of SSCs. The amount of SSCs expressing both GFRα1 and PLZF was reduced significantly (Fig. 4a, e, f) indicating that *Pten* played a crucial role in SSC self-renewal in the neonatal testis. To further reveal the molecular mechanisms accounting for the phenotype of this *Pten*-knockout testis, we used whole transcriptome sequencing to compare the gene expression profiles between wild type and *Pten*-knockout testes at 32 days old. Interestingly, we found that the expression of UTF1 significantly increased in the *Pten*-knockout testes (Additional file 1: Figure S1B). Next, immunostaining of the cross sections confirmed the increase of UTF1-expressing but PLZF-negative cells in *Pten*^−/−^ 7 day-old testis (Fig. 4b, g). At the age of day 10, similar to that of day 7, SSCs expressing GFRα1 or PLZF decreased (Fig. 4c, h, i). However, the number of UTF1^+^/PLZF^−^ cells was significantly higher in the *Pten*-knockout testes (Fig. 4d, j). As a chromatin-associated protein, UTF1 is involved in the initiation of ES cell differentiation [15]. In the testis, it has been evidenced that UTF1 is expressed in a subset of spermatogonial cells and germ cell neoplasms, making the SSCs maintain the ability of differentiation [4, 16]. In *Pten*^−/−^ mouse, we have seldom observed neoplasms in the testes. A number of UTF1^+^/PLZF^−^ cells and few UTF1^−^/PLZF^+^ cells were observed in the cross sections of 32 day-old *Pten*^−/−^ mouse testis by immunostaining (Additional file 1: Figure S1A), which implied that *Pten*-deletion-induced UTF1 expression might boost SSCs differentiation resulting in testes overgrowth in *Pten* knockout males.

### The PTEN signaling regulated the expression of PLZF and UTF1 in SSCs

**Fig. 5:**
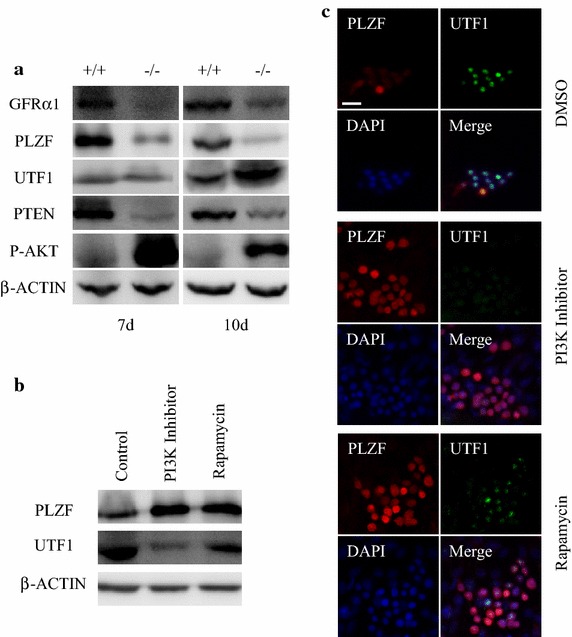
PLZF and UTF1 are regulated by the PI3K/PTEN signal pathway. **a** Western blot analysis of GFRα1, PLZF, UTF1, PTEN, and P-AKT expression in *Pten* ^+/+^ and *Pten* ^−/−^ testes at 7 and 10 days old. **b** Western blot analysis of PLZF and UTF1 expression in cultured SSCs isolated from 7 day-old testes. The treatment with PI3K inhibitor or rapamycin lasted for 96 h, DMSO was used as control. **c** Coimmunofluorescent staining of PLZF and UTF1 in SSCs treated with PI3K inhibitor, rapamycin or DMSO, showed that the PI3K signal pathway inhibition induced PLZF but reduced UTF1 expression (*scale bar* is 20 µm).

We further examined the expression of GFRα1, PLZF and UTF1 in *Pten*-deleted testes at day 7 or day 10 using Western blot analysis. Consistent with the data from cross section immunostaining, the expression of GFRα1 and PLZF was reduced at both day 7 and day 10, while UTF1 expression was highly increased at day 10 (Fig. 5a). As the PI3K/AKT/mTOR pathway is an important signaling pathway in regulating the cellular functions, PTEN is a major negative regulator of PI3K signaling. Then, we isolated SSCs from 7 days old pups and cultured them in the presence or absence of PI3K inhibitor or mTOR inhibitor-rapamycin to detect the role of PTEN signaling in SSCs in vitro. We found that PLZF expression was induced by inhibiting the PI3K signaling (Fig. 5b, c). However, in the same cell pool, UTF1 expression was reduced in the presence of PI3K inhibitor or rapamycin (Fig. 5b, c), implying the presence of a reversal relationship between PLZF and UTF1 in the context of the PTEN/PI3K signaling. Furthermore, UTF1 expression was slightly higher with Rapamycin than with PI3K inhibitor, which indicated that other branched pathways, in addition to mTOR signaling, may participate in this regulation. As we all know, PTEN is an inhibitor of PI3K signaling. Thus, we proposed that the PLZF and UTF1 expression was regulated by *Pten* in SSCs and *Pten* deletion led to reduction in PLZF expression associated with the increase of UTF1 expression.

### A putative model of SSC subpopulation fate controlled by *Pten*

**Fig. 6:**
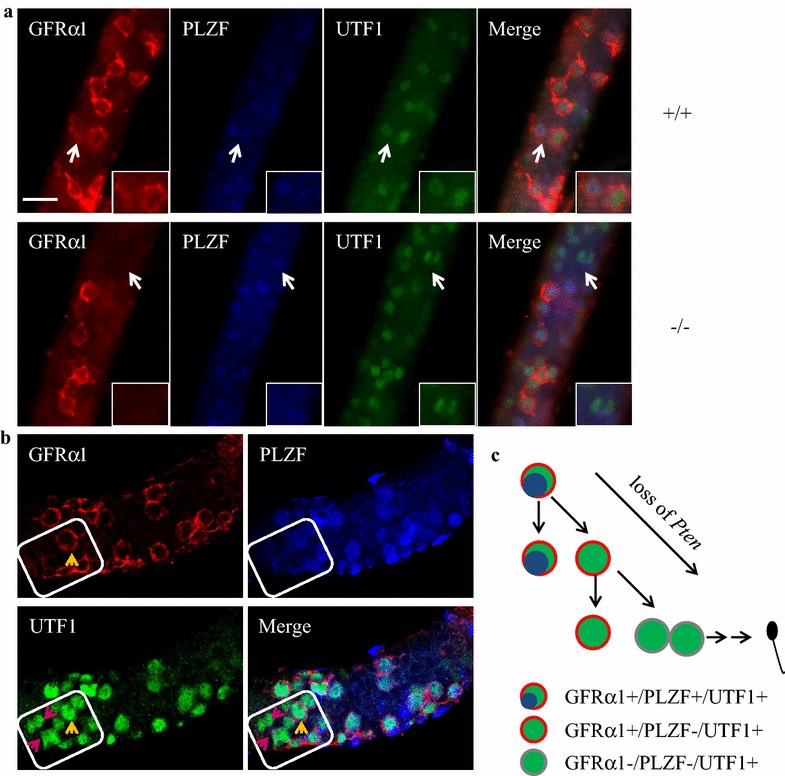
Loss of *Pten* triggers asymmetric division and increases the differentiation-associated proliferation of SSCs. **a** Whole-mount triple-immunofluorescence of GFRα1, PLZF and UTF1 in seminiferous tubules at 7 days old. In the *Pten* ^+/+^ tubules (*upper panel*), a pair of SSCs (*arrows*) consisting of one GFRα1^+^/PLZF^+^/UTF1^low^ cell and the other GFRα1^+^/PLZF^−^/UTF1^+^ (with UTF1 on the chromatin, *arrow*) cell was observed, which may undergo an asymmetric division of the stem cell. Meanwhile, a pair of GFRα1^−^/PLZF^−^/UTF1^+^ progeny (*arrows*) with UTF1 on chromatin was observed in the completing division in the *Pten* ^−/−^ seminiferous tubules (*lower panel*) (*scale bar* is 20 µm). **b** Whole-mount triple-immunofluorescence of GFRα1, PLZF and UTF1 in *Pten* ^−/−^ seminiferous tubules at 7 days old showing a group (marked in *square*) of GFRα1^−^/PLZF^−^/UTF1^+^ cells (*purple arrow*) next to GFRα1^+^/PLZF^−^/UTF1^+^ cells (*yellow arrow*) within the context of GFRα1^+^/PLZF^+^/UTF1^+^ SSCs. **c** A schematic diagram showing a hypothesis of asymmetric division of SSC in the testis at 7 days old. GFRα1^+^/PLZF^+^/UTF1^+^ stem cell underwent a division and gave rise to a GFRα1^+^/PLZF^−^/UTF1^+^ progeny, which in turn divided into two GFRα1^−^/PLZF^−^/UTF1^+^ cells towards differentiation fate. Loss of *Pten* accelerated this pathway and disturbed the balance of self-renewal versus differentiation of GFRα1^+^/PLZF^+^/UTF1^+^ stem cells, thereby causing SSCs depletion and infertility with age.

To further identify the properties of UTF1^+^, PLZF^+^ and GFRα1^+^ cells, we performed whole mount staining in 7 day-old tubules to locate their expression in SSC population. As shown in Fig. 6a, although most UTF1 positive cells are positive to both GFRα1 and PLZF, a subpopulation of UTF1^+^/GFRα1^+^/PLZF^−^ SSCs is present. UTF1 protein, which is revealed as a chromatin-associated protein [15], loads on chromosomes in GFRα1^+^/PLZF^−^ SSCs (Fig. 6a, upper panel). We observed the dividing spermatogonial cells expressing UTF1 but neither GFRα1 nor PLZF in the 7 day-old testicular tubules of *Pten*^−/−^ males (Fig. 6a, bottom panel). Furthermore, we observed the groups of GFRα1^−^/PLZF^−^/UTF1^+^ cells located adjacent the cells of GFRα1^+^/PLZF^−^/UTF1^+^ in the *Pten*^−/−^ 7 day-old testicular tubules (Fig. 6b). Therefore, we hypothesize a model of SSC fate: A GFRα1^+^/PLZF^+^/UTF1^+^ SSC may undergo asymmetric division, then generate a GFRα1^+^/PLZF^+^/UTF1^+^ cell for self-renewal and another GFRα1^+^/PLZF^−^/UTF1^+^ cell. The latter cell enters cell cycle, loses GFRα1 expression, and later develops into differentiating SSCs (Fig. 6c).

## Discussion

Prior to undergoing differentiation of meiosis, spermatogonial stem cells proliferate and form a pool of cells at different division status to meet the dynamics of spermatogenesis. This cell pool is maintained by self-renewal and proliferation of SSCs and exists throughout life time. The transcriptional factor PLZF plays a crucial role in the maintenance of SSC pool in adult males, and PLZF knockout causes a progressive loss of spermatogonia with age [1, 2]. Interestingly, *Pten* knockout male pups have less PLZF positive SSCs even at 7 day-old (Figs. 4a, f). We have reported that PLZF expression is regulated by the PTEN signaling pathway in prostate cells [17]. Similarly, PLZF expression was significantly reduced in the *Pten* knockout SSCs in this study (Fig. 5a), and further experiments in vitro confirmed that in SSCs, PLZF was indeed regulated by the PTEN signaling (Fig. 5b). Thus, in *Pten*^−/−^ males, the loss of SSCs is partly due to the reduction of PLZF expression.

The precise regulation of the balance of self-renewal versus differentiation of stem cells is critical in controlling tissue homeostasis and function. Excessive differentiation-associated proliferation leads to depletion of stem cells and degeneration of tissue with age. In *Pten*^−/−^ males, although the number of SSCs decreased, which occurred as early as 7 days after birth, testes underwent overgrowth or premature of larger size until day 42 but shrank afterward. This phenotype is apparently associated with excessive differentiation-proliferation of SSCs, which disturbs the long-term maintenance of stem cell pool, thereby leading to the exhaustion of spermatogenesis with age. Furthermore, *Pten* knockout induced the expression of UTF1, which is expressed in a subpopulation of spermatogonial cells in the testis [4, 16]. UTF1^+^ cells were significantly increased in the testis as early as at day 10 in the *Pten*^−/−^ males compared with wild-type males (Fig. 4d). Using immunostaining of a nearly infertile 32 day-old *Pten*^−/−^ testis, many UTF1^+^/PLZF^-^ cells and few UTF1^−^/PLZF^+^ cells (Additional file 1: Figure S1A) were observed, indicating that *Pten*-deletion-induced UTF1 expression might boost SSCs differentiation associated with the testes overgrowth of *Pten* knockout males. Moreover, it has been reported that UTF1 makes the spermatogonia maintain the ability of differentiation [4] and is involved in the initiation of ES cell differentiation [15]. All of these results indicate that UTF1^+^ cells are differentiating SSCs. To further identify the properties of UTF1^+^ cells, we performed a three color whole-mount staining of UTF1 with GFRα1 and PLZF of 7 day old tubules to locate UTF1 expression in the SSC population. Notably, a subpopulation of UTF1^+^/GFRα1^+^/PLZF^−^ SSC and UTF1^low^/GFRα1^+^/PLZF^+^ cell was observed, which seemed to come from the same precursor cell through asymmetric division. Similar subpopulation cells in *Pten*^−/−^ tubule lost both GFRα1 and PLZF expression. Therefore, we hypothesized a model of SSC fate in Fig. 6c. Thus, in this study, *Pten* knockout induced UTF1 expression in addition to causing the loss of PLZF expression. However, further studies should be conducted to address the mechanism how *Pten* regulates PLZF and UTF1 expression.

Although the testes overgrowth in *Pten* knockout males occurred within the first 2 months, the fertility and embryos production were lower compared with wild type males at same ages (Fig. 2b). Apparently, this phenotype is associated with the abnormality of sperms found in the epididymis which lack tails (Fig. 3b). This abnormality may be caused by the differentiation defects before haploid stages or during spermatogenesis because *Pten* is actively expressed in the haploid cells in the testis (Fig. 1d).

Collectively, The *Pten*-deletion-induced reduction of PLZF and increased expression of UTF1 apparently disturb the balance of self-renewal and differentiation of SSCs, leading to the depletion of spermatogonial cells and infertility with age.

## Conclusion

By studying the model of *Pten* knockout in SSCs, we found that *Pten* is required for the long-term maintenance of SSCs and spermatogenesis. Our study provides a new insight into the precise mechanisms controlling SSC self-renewal versus differentiation to maintain SSC pool and spermatogenesis throughout life time, especially the discovery of a *Pten*-regulated GFRα1^+^/PLZF^−^/UTF1^+^ progenitor population might lead to a new understanding of SSC fate control.

## Methods

### Animals

*Stra8*-cre mice (Stock number 008208) and *Pten*^LoxP/LoxP^ mice (Stock number 006440) were purchased from the Jackson Laboratory. *Stra8*-cre males were crossed with *Pten*^f/f^ females to generate *Pten* knockout in SSCs. Genotype of *Stra8*-cre mice and *Pten*^f/f^ mice were determined by PCR analysis using the primers and procedures provided by the Jackson Laboratory or by a previous research [18]. For *Pten* PCR, the *Pten*^f/f^ (1.1 kb) and *Pten* (1 kb) fragments were amplified by using the following primers: 5′-ACTCAAGGCAGGGATGAGC-3′ (forward), 5′-AATCTAGGGCCTCTTGTGCC-3′ (reverse). For *Stra8*-cre PCR, the *Stra8*-cre (179 bp) and *Interleukin 2* (*Il2* internal positive control, 324 bp) fragments were amplified by using primers: 5′-GTGCAAGCTGAACAACAGGA-3′ (*Stra8*-cre forward), 5′-AGGGACACAGCATTGGAGTC-3′ (*Stra8*-cre reverse); 5′-CTAGGCCACAGAATTGAAAGATCT-3′ (*Il2* forward), 5′-GTAGGTGGAAATTCTAGCATCATCC-3′ (*Il2* reverse). Animals used in this study were maintained according to the Guide for the Care and Use of Laboratory Animals (Publication 85-23, revised 1996; National Institutes of Health, Bethesda, MD, USA), and the protocol was approved by Shanghai Jiao Tong University School of Medicine (Shanghai, China)

### Histological analysis and immunostaining

Testes and epididymis were fixed in fresh Bouin’s fixative, embedded in paraffin and sectioned at 4 μm thickness. After the hematoxylin and eosin staining, the sections were mounted and viewed under a microscope (Carl Zeiss, Maple Grove, MN, USA).

For immunohistochemical staining, testes were fixed in 4% paraformaldehyde, embedded in OCT and sectioned at 8 μm thickness. The endogenous peroxidase activity was blocked by placing the slides in 3% hydrogen peroxidase for 10 min followed by a tap water rinse. After being blocked with 5% BSA, slides were subsequently incubated with the primary antibody against PTEN (1:50 dilution, BOSTER BA1377) at 4°C overnight, slides were then incubated with Biotin conjugated secondary antibody. Following incubation with Streptavidin-Biotin Complex (BOSTER SA1022), visualization was performed with a DAB reaction, thereby resulting in brown staining of structures containing the epitope. Cellular nuclei were counterstained with hematoxylin and slides were permanently mounted and evaluated under a light microscope. For immunofluorescent staining, after blocking with 2% BSA, frozen slides or cell slides were stained with antibodies against PLZF (1:100 dilution, R&D, AF2944), UTF1 (1:1,000 dilution, ABCAM, ab24273) or GFRα1 (1:40 dilution, R&D, AF560). The primary antibodies were revealed with Alexa-555 and Alexa-488 conjugated secondary antibodies together with DAPI to stain the nuclei. The sections were mounted and viewed under a fluorescence microscope. For statistical analysis, five different slides from *Pten*^+/+^ or *Pten*^−/−^ mice were stained and positive cell numbers were calculated and analyzed by one-way ANOVA (α = 0.05). For whole-mount staining, with enzymatic dissociation of the testes using 1 mg/ml collagenase for 5 min at 37°C, untangled seminiferous tubules were fixed with 2% paraformaldehyde containing 0.5 mM CaCl_2_ for 30 min at room temperature. After incubation with 1% Ttriton 100 for 10 min, samples were dehydrated through a series of methanol (25, 50, 75, and 100% in PBS containing 0.5% Triton 100—PBS-T) on ice followed by rehydration in PBS-T. The seminiferous tubules were incubated in a blocking buffer (1% BSA and 4% donkey serum) for 1 h and incubated with the first antibody combination at 4°C overnight. The appropriate second antibodies (Alexa-555, Alexa-488 and Dylight-405 conjugated) were applied onto the samples at room temperature for 2 h. After washing with PBS-T, the samples were mounted and observed under a fluorescence microscope.

### Western blot analysis

The proteins were extracted from the cells or testes using the lysis buffer containing 50 mM Tris-HCl (pH7.4), 1 mM EDTA, 150 mM NaCl, 1% sodium deoxycholate, 0.1% SDS, 10 mM sodium fluoride, 1 mM sodium orthavanadate and 1% protease inhibitor cocktail (Sigma-Aldrich Corp, St. Louis, MO, USA). The extracted samples containing 50 μg proteins were subjected to 10%SDS-PAGE and electrophoretically transferred to polyvinylidene difluoride membranes. The filter was probed with PLZF antibody (1:200 dilution, R&D AF2944), UTF1 antibody (1:250 dilution, Chemicon MAB4337), PTEN antibody (1:1,000 dilution, Millipore 04-035), GFRα1 antibody (1:2,000 dilution, R&D AF560), P-AKT antibody (1:1,000 dilution, Cell Signaling Technology #4058s) and β-actin (Cell Signaling Technology). Appropriate secondary antibodies were used and the antibody-antigen complexes in the membranes were visualized using an enhanced-chemiluminescent detection kit (Millipore). The images were scanned using LAS-4000 mini (FUJIFILM, Minato-ku, Tokyo, Japan).

### RNA isolation and RT-PCR analysis

The total RNAs were extracted using TRIzol reagent (Invitrogen) and then the RNAs were reverse transcribed by using a Reverse Transcription kit according to manufacturer’s instructions (TaKaRa, DRR037A). The following primers were used for SYBR Green–based real-time PCR (TaKaRa, DRR420A) on a 7900HT Real Time PCR System (Applied Biosystems Inc, USA): *Gapdh* [GenBank: NM_008084.3], 5′-TGCCCCCATGTTTGTGATG-3′ and 5′-TGTGGTCATGAGCCCTTCC-3′; *Pten* [GenBank: NM_008084.3], 5′-TTCATACCAGGACCAGAGGA-3′ and 5′-TTGTCATTATCTGCACGCTCT-3′. Relative gene expression was calculated by the two DDCt method against internal reference gene of glyceraldehyde-3-phosphate dehydrogenase (*Gapdh*).

### In vivo fertility assay

To evaluate the effect of *Pten*^−/−^ on fertility, we carried out in vivo fertility assay. For each experiment, two normal female mice were mated with one *Pten*^+/+^ or *Pten*^−/−^ male for 2 weeks and then embryos were counted. This mating test was artificially divided into three groups according to the male’s ages as follows: 35–60 days, 61–100 days, and older than 100 days. All statistical analyses were conducted with GraphPAD 5.0.

### Isolation of haploid cells

Testes were cut into pieces after removing the tunica albuginea, and testicular fragments in PBS were shocked roughly to wash out the intermediate cells near the lumen. Subsequently, the cells in supernatant were collected and stained with Hoechst 33342 (5 μg/ml). After 90 min of incubation, cells were resuspended in an ice-cold cell solution (PBS with 10% FBS) containing 2 μg/ml of propidium iodide for dead cell discrimination. All the solutions contain verapamil (50 μM/ml) to block the efflux of Hoechst. Finally, sorting was performed on an Influx cell sorter with UV laser (BD Biosciences) [19].

### Isolation and culture of spermatogonial stem cells

Testes were removed from pups with fine forceps using sterile procedures and cut into pieces after removing the tunica albuginea. Following a two-step enzymatic digestion at 37°C until the tubules became minimum, supernatants were pipetted and collected quickly. The supernatant was centrifuged to remove the collagenase and the cells were incubated in a dish for 1 h, when the somatic cells had adhered to the bottom of the dish, the supernatants were collected and resuspended in KO-DMEM medium containing 1% FBS and 1,500 units/ml LIF to 6 well plates (for western blotting) or to 12 well plates with covers in each well (for immunofluorescent staining). Recombinant human GDNF and bFGF were added at a final concentration of 20 and 1 ng/ml respectively. Cells were maintained at 34°C in a humidified 5% CO_2_ atmosphere [20]. The medium (containing 5 μM PI3K inhibitor or rapamycin 20 nM and growth factors) were changed every other day.

## Additional file

Additional file 1:
**Figure S1.** (A) Coimmunofluorescent staining of PLZF and UTF1 in 32 day-old *Pten* ^−/−^ cross sections showing a number of UTF1^+^/PLZF^−^ cells and few UTF1^−^/PLZF^+^ cells. (scale bar is 50 µm). (B) Whole transcriptome sequencing result of 32 day old *Pten* ^+/+^ and *Pten* ^−/−^ testes showing that UTF1 expression was significantly increased in the *Pten* ^−/−^ testes.

## Abbreviations

PTEN: phosphatase and tensin homolog
PLZF: promyelocytic leukaemia zinc finger
UTF1: undifferentiated embryonic cell transcription factor 1
PI(3)K: phosphatidylinositol-3-OH kinase
SSCs: spermatogonial stem cells
Stra8: stimulated by retinoic acid gene 8
EGFP: enhanced green fluorescent protein
GFRα1: GDNF family receptor alpha-1
FOXO3A: forkhead box O3
Il2: interleukin 2
LIF: leukemia inhibitory factor
FBS: fetal bovine serum
GDNF: glial cell-derived neurotrophic factor
bFGF: basic fibroblast growth factor
