# Supplementary figures and images for: PTEN signaling is required for the maintenance of spermatogonial stem cells in mouse, by regulating the expressions of PLZF and UTF1
Source: Cell Biosci. 2015 Jul 28;5:42. doi: 10.1186/s13578-015-0034-x (PMC4517568; doi:10.1186/s13578-015-0034-x)

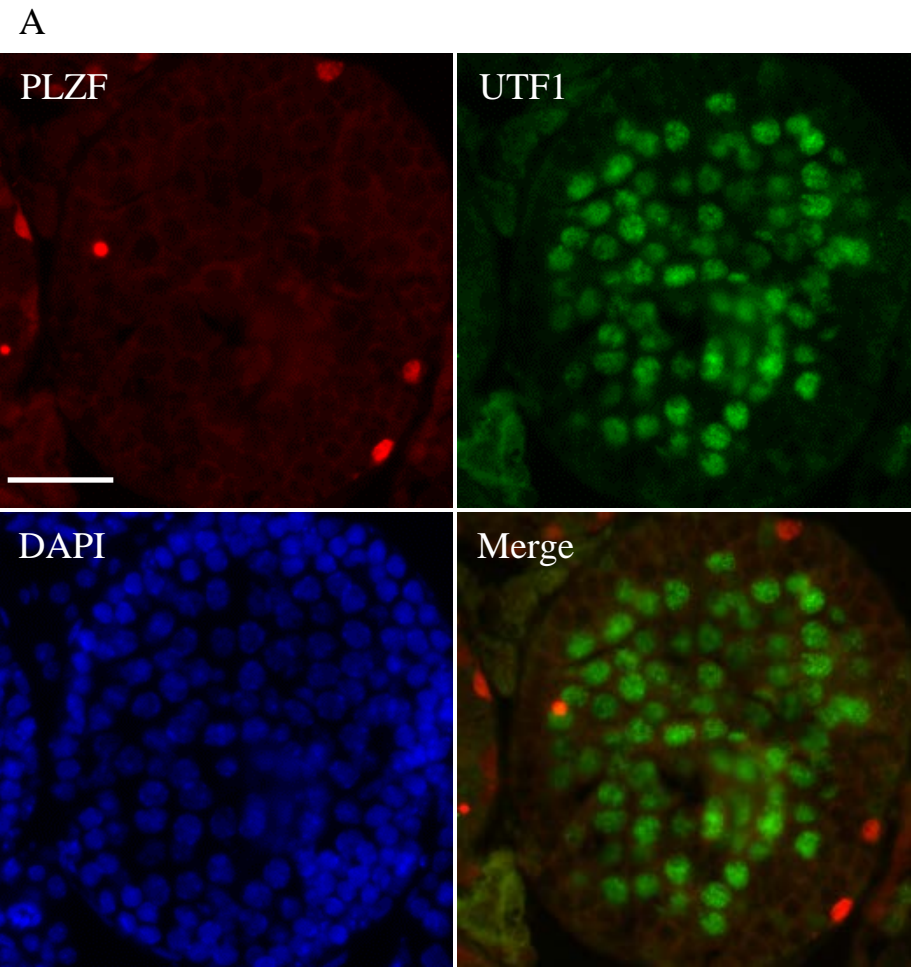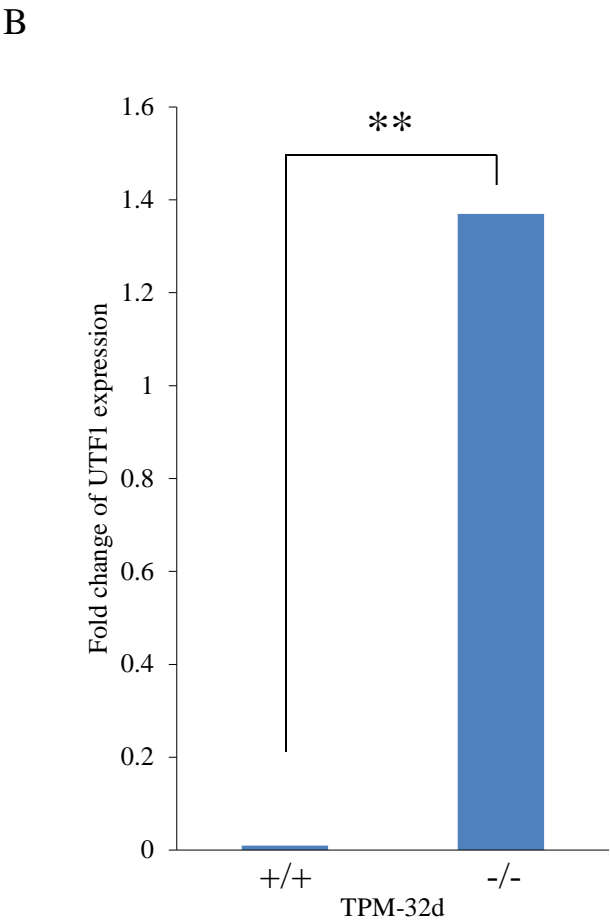

Supplement: Additional file 1: — Figure S1. (A) Coimmunofluorescent staining of PLZF and UTF1 in 32 day-old Pten −/− cross sections showing a number of UTF1+/PLZF− cells and few UTF1−/PLZF+ cells. (scale bar is 50 µm). (B) Whole transcriptome sequencing result of 32 day old Pten +/+ and Pten −/− testes showing that UTF1 expression was significantly increased in the Pten −/− testes. [file 13578_2015_34_MOESM1_ESM.pdf]
